# Metabarcoding analysis of strongylid nematode diversity in two sympatric primate species

**DOI:** 10.1038/s41598-018-24126-3

**Published:** 2018-04-12

**Authors:** Barbora Pafčo, Dagmar Čížková, Jakub Kreisinger, Hideo Hasegawa, Peter Vallo, Kathryn Shutt, Angelique Todd, Klára J. Petrželková, David Modrý

**Affiliations:** 1 Department of Pathology and Parasitology, Faculty of Veterinary Medicine, University of Veterinary and Pharmaceutical Sciences Brno, Palackého tř. 1946/1, 612 42 Brno, Czech Republic; 2 The Czech Academy of Sciences, Institute of Vertebrate Biology, Květná 8, Brno, 603 65 Czech Republic; 3 Department of Zoology, Faculty of Science, Charles University, Viničná 7, Praha, 128 44 Czech Republic; 4 Department of Infectious Disease Control, Oita University School of Medicine, 1-1 Idaigaoka, Hasama, Yufu, Oita, 879-5593 Japan; 5 Fauna & Flora International, Pembroke St, Cambridge, CB2 3QZ United Kingdom; 6 WWF-CAR, Bangui, Central African Republic; 7 The Czech Academy of Sciences, Institute of Parasitology, Branišovská 31, České Budějovice, 370 05 Czech Republic; 8 Central European Institute for Technology (CEITEC), University of Veterinary and Pharmaceutical Sciences Brno, Palackého tř. 1946/1, 612 42 Brno, Czech Republic

## Abstract

Strongylid nematodes in large terrestrial herbivores such as great apes, equids, elephants, and humans tend to occur in complex communities. However, identification of all species within strongylid communities using traditional methods based on coproscopy or single nematode amplification and sequencing is virtually impossible. High-throughput sequencing (HTS) technologies provide opportunities to generate large amounts of sequence data and enable analyses of samples containing a mixture of DNA from multiple species/genotypes. We designed and tested an HTS approach for strain-level identification of gastrointestinal strongylids using ITS-2 metabarcoding at the MiSeq Illumina platform in samples from two free-ranging non-human primate species inhabiting the same environment, but differing significantly in their host traits and ecology. Although we observed overlapping of particular haplotypes, overall the studied primate species differed in their strongylid nematode community composition. Using HTS, we revealed hidden diversity in the strongylid nematode communities in non-human primates, more than one haplotype was found in more than 90% of samples and coinfections of more than one putative species occurred in 80% of samples. In conclusion, the HTS approach on strongylid nematodes, preferably using fecal samples, represents a time and cost-efficient way of studying strongylid communities and provides a resolution superior to traditional approaches.

## Introduction

The order Strongylida represents one of the major radiations of nematodes, involving a vast diversity of mammalian gastrointestinal parasites, including important human pathogens. Strongylid community diversity and abundance depend on a range of factors and differ significantly between mammalian taxa^1^. Strongylid nematodes form complex communities in large terrestrial herbivores, such as great apes^2^, equids^3^, artiodactyls^4^ and elephants^5^. However, identification of individual strongylid species occurring in complex communities using traditional methods based on coproscopy, followed by microscopy or single nematode amplification and Sanger sequencing, is time-consuming and lacks sensitivity to detect low abundance species. Moreover, neither the basic coproscopy methods nor more advanced analyses of larval morphology/morphometry allow robust species-level determination. At the same time, Sanger sequencing methods, which offer higher discriminative power, can become quite expensive^6,7^.

A range of high-throughput sequencing (HTS) technologies provides an opportunity to generate large amounts of sequence data in a short time and at low cost^6,7^, allowing examination of samples containing a mixture of DNA from multiple species/genotypes of the studied organisms where even rare taxa can be captured. Thus, such approaches represent a powerful tool for analyses of complex multi-species communities. Over the past decade, numerous metabarcoding analyses of complex bacterial profiles of host microbiota have been carried out, based on HTS using 16S ribosomal RNA gene^8–14^. Also, studies on eukaryotic diversity employing HTS have described in detail complex planktonic and biofilm microbial populations^15–17^, free living protists^18^ and communities of free-living and plant parasitic nematodes^19,20^. However, the use of amplicon HTS for the evaluation of complex metazoan parasite communities remains significantly underexplored^21–24^. A few previous studies have used 18S rDNA HTS profiling for the description of gut nematode communities in various vertebrate hosts^21,24^. However, as evolutionary rates of eukaryotic 18S rRNA gene are rather low, these studies provide only superficial taxonomic resolution, which may consequently conceal important factors affecting assembly patterns of gut nematode community.

Strongylid nematodes are among the most common gut parasites of non-human primates (NHP)^25–29^, and can have adverse effects on health status and fitness of great apes^30,31^. Moreover, the close phylogenetic relationship between humans and NHPs results in a partial overlap in their pathogens and strongylid transmission from humans to native populations of NHPs can potentially be of significant concern to their conservation. At the same time, NHPs can pose an important source of human parasites, including strongylid nematodes. Consequently, communities of strongylid nematodes hosted by NHPs have attracted significant attention from the scientific community^30–37^. Despite a number of important discoveries, previous studies on NHP strongylids were limited by: (i) an absence of reference sequences for most of the helminth taxa concerned, (ii) the inability to detect rare species, and, (iii) ubiquitous mixed infections by several strongylid taxa. Put in other words, although the traditional methods such as basic coproscopy, larval morphology/morphometry or Sanger sequencing are suitable for detection of dominant members of strongylid communities, their potential to determine specific and crucial information on strongylid ecology and epidemiology is rather limited. Such information includes the identification of the drivers influencing spatio-temporal variation in strongylid prevalence, coinfection patterns or between-host transmission.

Our aim was to develop a HTS approach that overcomes the limitations of current strongylid nematode research. Specifically, this method should (i) provide consistent strongylid community profiles, (ii) allow fine-scale taxonomic placement of detected haplotypes and (iii) be applicable to a broad scale of mammalian host species. The described methodology is based on the sequencing of ITS-2 amplicons. This taxonomic marker was selected as the majority of previous non-HTS studies relied on ribosomal internal transcribed spacer data^32,38,39^. Consequently, extensive reference data for ITS markers already exist. Importantly, the faster evolutionary tempo of ITS compared to 18S rRNA (another ribosomal DNA marker commonly used in molecular taxonomy) guarantees a finer taxonomic resolution of the resulting haplotypes. Also, the presence of highly conservative regions flanking ITS enables, unlike most protein-coding taxonomic markers, the design of conservative PCR primers, robust against allelic drop-out^40^. Moreover, in parallel, strongylid community profiles were obtained from (i) total fecal DNA, and, (ii) nematode larvae developed via coprocultures; approaches commonly used in strongylid parasite studies of humans, great apes and other vertebrates^41–43^. To our knowledge, this is the first study that provides relevant empirical data comparing the effectiveness of these methods.

We tested our approach on samples from sympatric populations of two free-ranging NHP species, namely western lowland gorillas *Gorilla gorilla gorilla* (Savage, 1847) and agile mangabeys *Cercocebus agilis* (Milne-Edwards, 1886) in Dzanga-Sangha National Park in Central African Republic. Despite broad similarities in their lifestyles (being predominantly terrestrial, living in social groups, sharing the same habitat), and frequent contact with humans, these two NHPs also exhibit pronounced differences in their ecological and life-history traits. Specifically, they differ in body size, group and home range size and dietary adaptations (mangabeys are more insectivorous and may occasionally hunt and feed on animal prey)^44^. Accounting data obtained using traditional parasitological approaches, we presumed that strongylid fauna of these two primate species partially overlap, making them a suitable model to test the applicability of a designed HTS barcoding approach.

## Results

### Method sensitivity and effectivity

After quality filtering we did not find any ITS-2 sequences in the negative controls (isolate of metagenomics DNA from an uninfected individual) and only haplotypes perfectly matching the artificial construct were recovered from the corresponding positive controls. This indicates that cross-contaminations between samples were unlikely to affect the observed haplotype overlapping among individuals. Lack of any unexpected haplotypes in positive controls revealed that our wet lab and bioinformatic pipeline is robust against PCR and sequencing artefacts. All three artificial haplotypes were recovered from positive controls irrespective of the dilution factor of the original mixture, implying that our method is sufficiently sensitive for the detection of <10 ITS-2 copies per PCR reaction. Despite differences in amplicon length, the average proportion of reads recovered for *Oesophagostomum* and *Necator* constructs was comparable (ANOVA: F_(1,14)_ = 0.839, p = 0.375; construct sequence mean *Necator americanus* (Stiles, 1902): 8,280 ± 677; *Oesophagostomum stephanostomum* Stossich, 1904: 8,192 ± 602; *Necator* sp. 6,250 ± 508)).

### Diversity of strongylids as revealed by HTS

**Figure 1:**
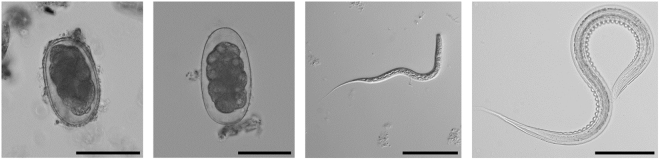
Strongylid nematodes found in studied DSPA primates, using coproscopy: two size categories of strongylid eggs (1; 2), L3 larva of *Oesophagostomum* (3) and L3 larva of *Necator* (4). Scale bars: 50 µm (1–2), 200 µm (3) and 100 µm (4).

All samples used were classified by coproscopy as positive for strongylid nematodes eggs; identification to genus level was performed based on the morphology of L3 larvae from coprocultures, revealing a 100% and 63% presence of *Necator* Stiles, 1903 and *Oesophagostomum* Molin, 1861 larvae, respectively (Fig. 1). Our sequencing data comprised 2,163,014 high quality reads that passed all filtering steps. In real samples, we detected 28 ITS-2 variants (haplotypes) that corresponded to four nematode genera: *Necator* (12 ITS-2 haplotypes, present in 80% of individuals) and *Oesophagostomum* (13 ITS-2 haplotypes, present in 80% of individuals) found in both gorillas and mangabeys, *Ternidens* Railliet & Henry, 1909 (1 ITS-2 haplotypes, present in 10% of individuals, corresponding to *Ternidens deminutus* (Railliet & Henry, 1905) NCBI accession: AJ888729, using blast searches against nt/nr NCBI database) and *Libyostrongylus* Lane, 1923 (1 ITS-2 haplotype, present in 5% of individuals, an identical match to the *Libyostrongylus* environmental sample, NCBI accession: JX159807) found in gorillas only.

### Comparison of results from coprocultures and feces

**Figure 2:**
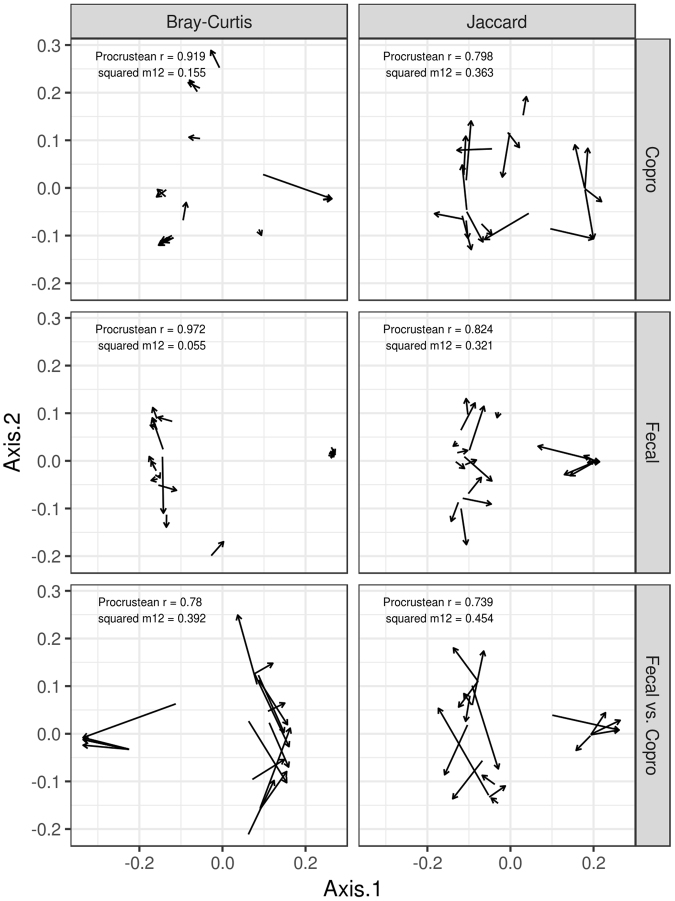
Consistency in strongylid community composition between PCR duplicates for (i) coprocultures (copro), (ii) feces (fecal) and (iii) coprocultures vs. feces from the same individual assessed via Procrustean superimposition running on Bray-Curtis and Jaccard dissimilarities. Procrustean correlation coefficients are shown. In all cases, associated permutation-based p values were <0.001.

**Figure 3:**
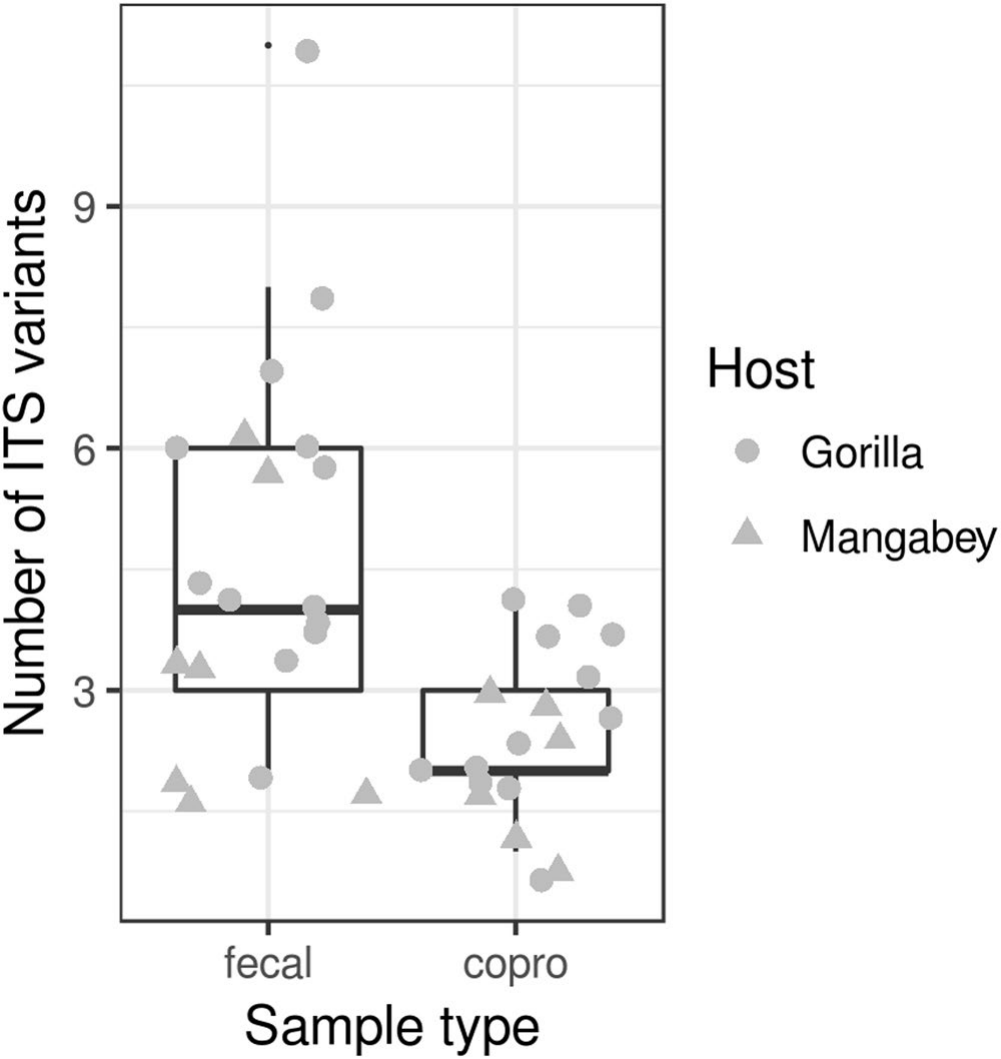
Box-plots of haplotype diversity represented by the number of all strongylid haplotypes detected in fecal samples (fecal) and coprocultures (copro) from DSPA gorillas and mangabeys.

Procrustean analyses performed for fecal samples and coprocultures revealed tight correlation between technical duplicates both in relative abundance and in presence vs. absence of all ITS-2 variants prior to filtering of putatively chimeric sequences (p < 0.0001 in both cases). Nevertheless, the higher concordance between fecal sample duplicates compared to coproculture duplicates is worth noting. This was supported by both methods employed, however, procrustean Bray-Curtis dissimilarities that put more weight on the most abundant ITS-2 variants, show stronger support than Jaccard dissimilarities that just account for presence vs. absence of any given ITS-2 variant, and thus also reflect rare or chimeric variants (Fig. 2). After elimination of putatively chimeric variants and sequence read counts merging among technical duplicates, there was a relatively tight correlation between fecal samples and coprocultures from the same individual (Fig. 2, p < 0.0001). GLMMs on the number of ITS-2 variants detected in individual samples showed that the coprocultures underestimated total haplotype diversity compared to fecal samples (Fig. 3, GLMM: estimate = −0.625 ± 0.182, ΔD.F. = 1, χ^2^ = 12.549, p = 0.0004).

### Comparison of strongylid communities of gorillas and mangabeys

**Figure 4:**
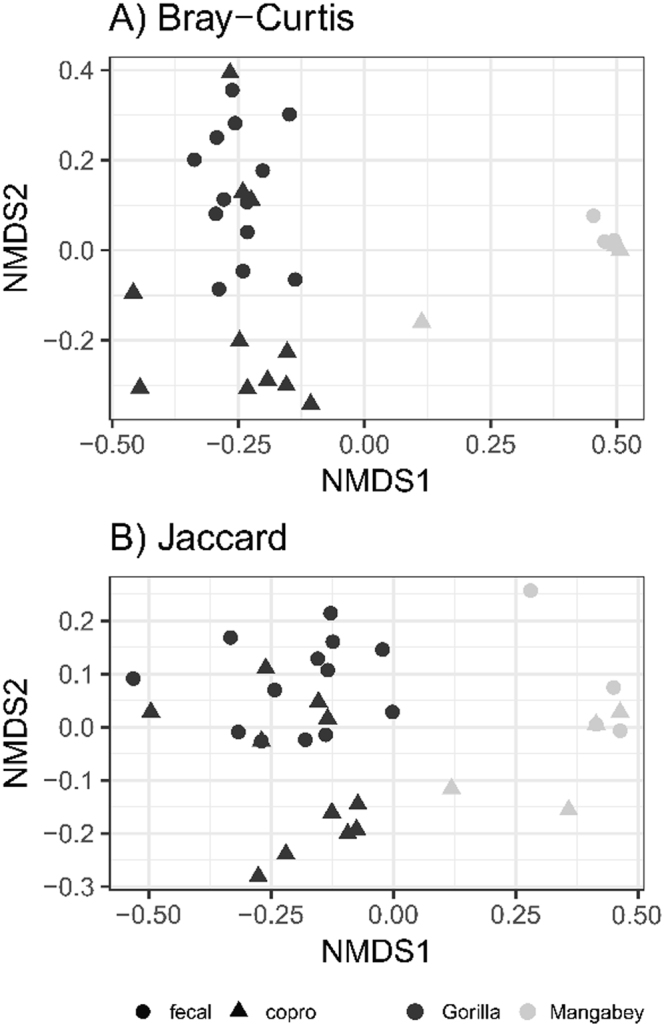
Non-metric Multidimensional Scaling (NMDS) for between-sample differentiation calculated based on relative haplotype abundances of all strongylid nematodes detected in fecal samples (fecal) and coprocultures (copro) from gorillas and mangabeys in DSPA.

Accounting for systematic differences in the number of ITS-2 variants recovered from fecal samples vs. coprocultures, gorilla nematode diversity was higher than that in mangabeys (GLMM: estimate = −0.406 ± 0.185, ΔD.F. = 1, χ^2^ = 5.053, p = 0.0246). Adonis and Non-metric Multidimensional Scaling showed that strongylid nematode community profiles from both fecal samples and coprocultures clustered according to host species identity (Fig. 4, Adonis: F_(1,35)_ = 37.982, R^2^ = 0.482, p = 0.001 for Bray-Curtis and F_(1,35)_ = 18.787, R^2^ = 0.332, p = 0.002 for Jaccard dissimilarities). Additionally, gorilla fecal samples and coprocultures tended to be separated along the second ordination axis (Fig. 4, Adonis: F_(1,35)_ = 5.833, R^2^ = 0.074, p = 0.001 for Bray-Curtis and F_(1,35)_ = 2.839, R^2^ = 0.050, p = 0.002 for Jaccard dissimilarities).

Strongylid community divergence between gorillas and mangabeys was driven by changes in the relative abundance reads corresponding to *Oesophagostomum* spp. vs. *Necator* spp., where the average proportion of reads corresponding to *Oesophagostomum* spp. was only 0.465 in gorillas compared to 0.862 in mangabeys (Fig. S1, GLMM: ΔD.F. = 1, χ^2^ = 13.512, p = 0.0002). In addition, there was a lower proportion of *Oesophagostomum* spp. reads recovered from coproculture vs. fecal sample sequencing when testing for all samples (0.472 vs. 0.756; GLMM: ΔD.F. = 1, χ^2^ = 7.043, p = 0.008).

We also observed considerable variation in strongylid community structure between the two hosts at the level of individual ITS-2 variants. From thirteen *Oesophagostomum* ITS-2 haplotypes three were found in both hosts, five solely in gorillas and five solely in mangabeys where only members of *O*. *stephanostomum*/*bifurcum* cluster were detected. Interestingly, according to our phylogenetic reconstruction, *Oesophagostomum bifurcum* (Creplin, 1849) represents a paraphyletic group, which hindered clear species-level assignation of *Oesophagostomum* haplotypes detected in our samples. Despite this fact, clear host-specific abundance variation patterns were observed. In particular, haplotype H3 that clustered with the LC063697 *O*. *stephanostomum* reference was present in almost all gorilla samples, but not in mangabeys. On the other hand, haplotype H2 was dominant in mangabeys but was nearly absent in gorillas. This haplotype forms, together with other *Oesophagostomum* haplotypes detected in our samples, a distinct monophyletic cluster. Unfortunately, we did not find any reference sequence belonging to this cluster, which precludes a clearer taxonomic delimitation.

**Figure 5:**
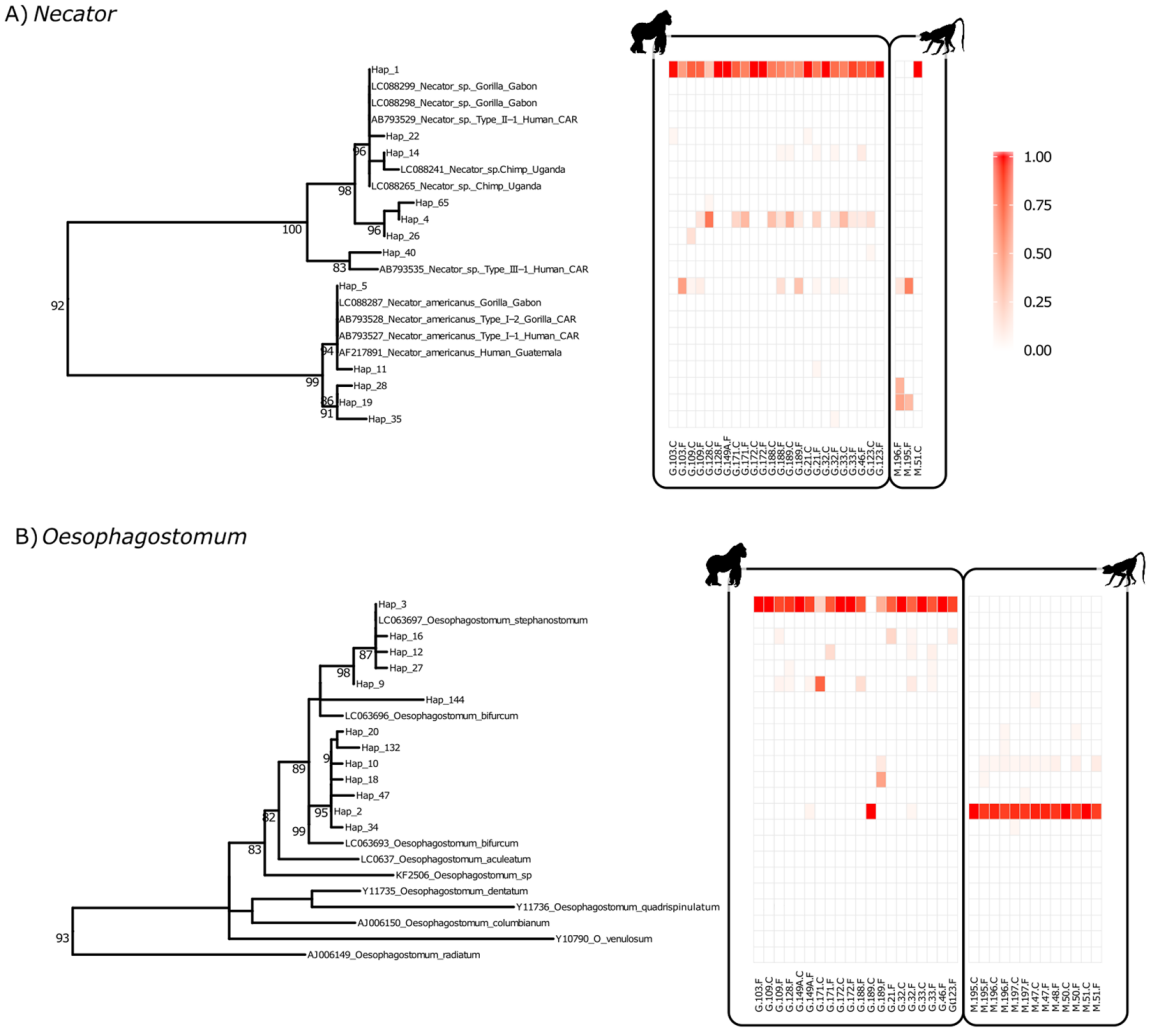
ML Phylogenetic tree for (**A**) *Necator* and (**B**) *Oesophagostomum* ITS-2 haplotypes in positive samples. Relative abundances of individual ITS-2 variants are indicated by color intensities in heatmaps. Bootstrap values greater than 80 are shown. The first letter in the sample code indicates species identity (M = mangabey, G = gorilla) and the last letter indicates sample type (F = fecal, C = coproculture).

In total, we found twelve haplotypes of *Necator* in both gorillas and mangabeys. Two of them were common in both hosts, eight were found solely in gorillas and two in mangabeys only. Phylogenetic analyses showed that the genus *Necator* formed two distinct clades, one grouped with the *N. americanus* sequences previously found in humans and gorillas in DSPA^33^, and a second *Necator* sp. clade previously found in primates in DSPA and in gorillas in Gabon^33,34^ (Fig. 5).

## Discussion

We designed and tested a high-throughput sequencing approach for strain-level identification of parasitic strongylid nematodes of non-human primates based on ITS-2 sequences, with the aim of developing a high-caliber research tool that can be used widely for studies of parasitic strongylid communities in various mammalian hosts including humans. Our meta-barcoding experiment required a sequencing technology with low error rates due to the need for fine-scale haplotype delimitation but allowed for relatively low-throughput due to amplicon sequencing and read lengths. Accounting for these requirements, Illumina pair-end sequencing with MiSeq platform was an obvious choice.

A growing number of reference sequences from identified strongylids in genomic databases provide an opportunity for strongylid determination. Both nuclear and mitochondrial genes are targeted in phylogenetic and taxonomic studies on strongylid nematodes^45^, however, ribosomal internal transcribed spacer (ITS-1 and ITS-2) sequences are most commonly used to discriminate among nematode species^32,38,39^. As the ITS regions are among the most variable nuclear loci with a sufficient number of comparative sequences in databases^45^, we used ITS-2 in our study. On the other hand, the rapid mode of ITS evolution including frequent indels, makes alignment of ITS haplotypes more challenging compared to protein-coding markers, which can adversely affect the reliability of phylogenetic reconstruction. Avramenko *et al*.^22^ as well as Lott *et al*.^23^ successfully employed the same locus for describing and quantifying species composition of parasitic nematode communities in cattle and red kangaroo using a HTS approach.

Strongylid nematode identification from animal feces depends on the investigation of either ova or developed L3 larvae. For example, in ruminant samples, larvae examination allows relatively precise species determination and quantification^43,46^. Recent studies on primate strongylid diversity using DNA-based techniques exploited DNA both from eggs^38,39^ and single larvae^33,34,42^. To test the impact of the type of material examined on ITS amplification and HTS sequencing outcomes, we isolated total DNA from fecal samples and, in parallel, from coprocultures developed from the same sample. We detected a higher diversity of strongylid profiles in fecal samples than in coprocultures. This apparent bias can be caused by the selective decline or losses of the eggs or larvae of a particular species during sample culturing due to differences in the ecological requirements of individual species or by inter-species interactions in the coprocultures^47–50^. Indeed, our data showed that in gorillas, the genus *Oesophagostomum* is far more evident using total DNA directly obtained from feces samples than DNA sourced from coprocultures. Despite the advantages of cultured larvae for strongylid taxonomy and the possibility of genetic analysis of single individuals, our results strongly favor fecal samples as a source of the DNA for HTS based metabarcoding.

Unlike previous studies describing strongylid nematodes and their transmission using classical chain-termination methods^33,35,39^, this is the first study attempting to describe complete strongylid community structures and the potential overlap between two sympatric NHP species. HTS applied in our study revealed a diversity of haplotypes, however, overall, the results remain consistent with previous studies. *Necator* and *Oesophagostomum*, the only genera detected based on larval morphology^27,51^, were the most prevalent strongylids in our dataset. The mean number of haplotypes present in a single sample was 4.7; however, the richest sample contained as many as 11 different haplotypes. In contrast to straightforward generic assignment, the interpretation of within-species ITS-2 haplotype diversity is more problematic. The total haplotype diversity of a single strongylid species can be partitioned to within-individual and between-individual variation. Within-individual diversity can arise due to heterozygosity of ITS-2 in diploid genomes and due to sequence polymorphism among rDNA paralogues^52^. Under the divergent paralogues scenario, some of the less represented haplotypes can actually belong to low-copy paralogues, while the dominant haplotypes such as H1 in *Necator* or H3 and H47 in *Oesophagostomum* can represent high-copy paralogues. Alternatively, if between-individual variation prevails, the less represented *Necator* and *Oesophagostomum* haplotypes could originate from less frequent taxa within these genera. To resolve this issue in future, we propose to apply the HTS approach to obtain ITS-2 haplotypes from single larvae, which will allow the determination of within and between-individual haplotype variability in the two most prevalent strongylid genera. Nevertheless, unlike traditional approaches, HTS also allowed the detection of rare, otherwise overlooked taxa such as *Ternidens diminutus* and *Libyostrongylus* sp. present in a small number of samples and represented by a low numbers of reads. There is a possibility that rather than *Libyostrongylus* sp. the latter taxon instead represents *Paralibyostrongylus* Ortlepp, 1939, of which sequence is missing in the databases.

Although *Necator* sequences were detected in all gorilla samples, their prevalence in mangabeys was much lower (43%). *Necator* ITS-2 haplotypes were clearly divided into two clades. The first clade can be referred to as *Necator americanus*, a species originally described in humans^53^ and also detected in NHPs^33,34,54^. In our study, we found *N*. *americanus* haplotypes both in gorillas and mangabeys. The second clade was comprised of non-*americanus* sequences type II and III, found both in gorillas and mangabeys, and described previously in humans and lowland gorillas from CAR and Gabon^33,34^. *Necator* type II was present in all gorillas, but also found in mangabeys. This putative species was recently assigned most probably to *Necator gorillae* Noda & Yamada, 1964 based on morphological determination of adult worms from the same locality^55^. *N*. *gorillae* was present in all gorillas, but also found in mangabeys. In our study, Type III, which was previously reported only in humans in CAR^33^, was found as a single haplotype in low prevalence in gorillas.

Analysis of *Oesophagostomum* sequences revealed the presence of two main haplotype groups. While the majority of sequences retrieved from gorilla samples clustered with the reference sequence of *O*. *stephanostomum*, a common species found in great apes across different regions^30,36,38,39,41^, most of the sequences originating from mangabeys formed separate clades. Whether we can refer to the clades as *O*. *bifurcum* or not, remains questionable. So far, *O*. *bifurcum* is the most commonly reported *Oesophagostomum* from African NHPs^39,56^, however, previously published analyses indicate the presence of more species within this group^36^. The distribution of detected *Oesophagostomum* haplotypes shows clear tendency towards host specificity, however, 3 out of 13 examined gorillas also hosted mangabey-type *Oesophagostomum*, indicating haplotype overlapping between the two NHP species.

Our HTS approach revealed co-occurrence of more than one haplotype in more than 90% of samples. Importantly, 80% of individuals were co-infected by more than one putative species. Our results proved that “strongylid eggs” reported in fecal samples of non-human primates by coproscopic tools represent a complex strongylid community of several species belonging to at least five different genera. In conclusion, high-throughput sequencing of strongylid nematodes from fecal samples represents a time- and cost-efficient way of studying helminth communities and provides a resolution superior to traditional approaches. Its application overcomes the limitations of classical Sanger sequencing and allows for analyses of strongylid nematode host-specificity in complex parasite-host systems. Although helminth communities are not as complex as bacterial ones, uncovering their diversity offers a yet unexplored opportunity to address interspecies interactions and complex epidemiology^22^.

## Material and Methods

### Samples available

Dzanga-Sangha Protected Areas (DSPA), Central African Republic (CAR) is the first and only site to have both lowland gorillas and agile mangabeys habituated for both ecotourism and research. We sampled these sympatric western lowland gorillas (*Gorilla gorilla gorilla*) and agile mangabeys (*Cercocebus agilis*) between July-September 2011. For this study we randomly selected 20 samples from different individuals which were represented by both a fecal sample and a coproculture developed from the same feces, thirteen from gorillas and seven from mangabeys inhabiting Dzanga-Ndoki National Park within DSPA. For further description of the field site and studied animals see Sak *et al*.^57^, Mapua *et al*.^58^ and Devreese^59^. We collected fresh feces immediately after defecation and/or the samples from fresh morning nests within three hours from the time we suspected the gorillas had left the nests. We mixed the internal content of the feces and fixed one part immediately in the field and collected the second part to develop the larvae. In our *in-situ* field lab, we implemented modified Harada-Mori fecal cultures^60^ to develop infectious L3 larvae from both gorilla and mangabey feces. We fixed both feces and developed L3 larvae in 4% formaldehyde for coproscopic/morphological examination and 96% ethanol for DNA isolation. All samples were collected non-invasively, adhering to site regulations regarding proximity to the animals and other health and safety protocols. All material was shipped to the Department of Pathology and Parasitology at UVPS, Brno, Czech Republic.

### DNA isolation and sequencing

We isolated the total genomic DNA from (i) fecal samples, and (ii) strongylid larvae developed by coprocultures from individual samples using respectively (i) PowerSoil kit (MO BIO Laboratories, Qiagen Company, USA) and (ii) Tissue genomic DNA mini kit (Geneaid Biotech Ltd., Taiwan), following the manufacturer’s protocols. We designed and optimized PCR protocols for amplification of the second internal transcribed spacer (ITS-2) of nuclear ribosomal DNA with forward primer Strongyl_ITS-2_F (acg tct ggt tca ggg ttg) and reverse Strongyl_ITS-2_R (atg ctt aag ttc agc ggg ta). We generated HTS sequencing libraries using a two-step-PCR approach following Fluidigm Access Array primer design. In the first PCR we used inner locus specific primers with “tails” serving as priming sites for the second PCR with outer PCR primers containing sample-specific barcodes and sequencing adaptors (i.e. Access Array Barcode Library for Ilumina Sequencers, Fluidigm Corporation, USA). We performed the first PCR using Kapa 2 G Robust Hot Star polymerase (Kapa Biosystems), under the following conditions: for the first step, 95 °C for 3 min, (95 °C 15 s, 55.5 °C 15 s, 72 °C 15 s) × 30, and 72 °C for 1 min; and for the second step, 95 °C for 3 min, (95 °C 15 s, 55 °C 30 s, 72 °C 30 s) × 16, and 72 °C for 3 min. We included a total of 40 DNA samples, using in parallel: all DNA isolated from feces (n = 20); and DNA isolated from a mixture of larvae from the coprocultures (n = 20) developed from the same fecal sample. Analyses were carried out in two technical replicates (duplicates) with different barcodes. We cleaned up the final library using the Agencourt AmpureXP beads (Beckman Coulter Life Sciences) and our target DNA size was selected using Pippin Prep (Sage Science, Inc., USA). We quantified the library using Kapa Library Quantification Kit (Kapa Biosystems) and sequenced using MiSeq Reagent Kit v2 (2 × 250 bp pair end reads) by Illumina MiSeq platform.

### Primer design and controls used during the PCR

We designed the primers manually based on the alignment of ITS-2 sequences corresponding to a broad range of strongylid nematodes (i.e. suborder Strongylida). We downloaded ITS-2 sequences for as many strongylid genera as possible from Genebank database. We aligned the sequences using MAFFT v. 1.3.5. and placed primers into conservative regions with a variable region in between. The alignment, with marked primer sites and filtered to include only sequences with the complete amplified region, is given in (Fig. S2, Alignment S3). For all downloaded sequences, the reverse or the forward primer showed maximum one mismatched base, which never occurred in the first six bases from the 3′ end. In addition, we did not observe any indels in priming sites, suggesting suitability of these primers for a broad range of strongylid taxa. Primer Blast analysis showed that occasional amplification may also occur outside of the Strongylida suborder (e.g. genera *Caenorhabditis* Osche, 1952, *Oscheius* Andrássy, 1976, *Panagrolaimus* Fuchs, 1930, *Steinernema* Travassos, 1927), but did not include other important parasitic nematodes such as members of the genus *Strongyloides* Grassi, 1879 (Rhabditida suborder). The primers amplify 349–359 bp and 241 bp of ITS-2 for *Necator* and *Oesophagostomum*, respectively.

During the PCR optimizations and also for PCRs included in the sequencing run, we used two negative controls, (i) DNA extraction from strongylida negative human feces and (ii) water. As positive controls and to test for biases (chimeric sequences, PCR errors, sequencing errors, contaminations) occurring during sample processing we used synthetic DNA templates (gBlocks Gene Fragments) carrying ITS-2 sequences of *Necator* sp., *Necator americanus* and *Oesophagostomum stephanostomum*. We created the three sequence constructs (see Appendix S4) based on sequences available in GenBank including ITS-2 of the strongylids species presumed present in the study samples based on previous analyses^37^ and results from the morphological determination of L3 larvae in coprocultures. For the purpose of later analyses, the final construct included 4 bp tags that did not match any real ITS-2 sequences. These constructs were mixed in equimolar ratios, to simulate mixed infections of strongylids in real samples, and diluted in DNA extraction from negative human feces to contain 999, 99 and 9 copies of ITS-2 sequences in the PCR template. We performed two independent PCRs on each dilution, which were then included into the sequencing run.

### Data processing and statistics

Raw fastq files were deposited into European Nucleotide Archive (project accession no.: PRJEB21189). Sample metadata along with sample accession numbers are given in Table S5. We demultiplexed resulting fastq files using skewer^61^ and assembled paired-end reads by pear^62^. Subsequently, using dada2^63^ we eliminated sequences, where the expected number of sequencing errors was higher than one and performed denoising on the filtered dataset to estimate relative abundances of ITS-2 haplotypes in individual samples. According to our analyses of “mock communities” (i.e. samples containing solely ITS-2 constructs), the default dada2 algorithm for chimera detection is associated with a considerable risk of false positives (i.e. real ITS-2 haplotypes being erroneously identified as chimeras). Consequently, assuming that the probability of independent occurrence of the same chimeric variant is low, we marked ITS-2 variants that were not consistently present in both technical duplicates, as putative chimeras or other kinds of non-biological variants. If not otherwise stated, our results are based on the dataset that does not include variants that differ between technical duplicates (i.e. putative chimeras and other kinds of PCR/sequencing artefacts) and where sequence counts for ITS-2 haplotypes consistently present in both duplicates were merged. Using blast searches against the nt/nr NCBI database, we performed taxonomic assignment of ITS-2 haplotypes up to genus level.

We employed Procrustean analysis (*A correlation technique for multivariate data*) to test for consistency in community composition between technical duplicates for (i) fecal samples and (ii) coprocultures as well as for (iii) consistency among fecal samples vs. coprocultures from the same animal. Binary Jaccard (accounting for presence vs. absence of individual ITS-2 variants) and Bray-Curtis dissimilarities (accounting for relative abundance of IT2-S variants) scaled by Principal Coordinate Analysis were used for consistency testing. Significance testing was based on a permutation procedure implemented in protest function (R package *vegan*). Furthermore, we reported a correlation-like statistic derived from the symmetric Procrustes sum of squares (hereafter “Procrustean r”) and the symmetric analysis sum of squares (hereafter “*squared m12*”). The Procrustean r increases with increasing concordance between two multivariate objects but the opposite is true in the case of *squared m12*^64^.

We compared the number of ITS-2 variants detected in fecal samples vs. coprocultures and in gorillas vs. mangabeys using Generalize Linear Mixed Effect Models (GLMM) with a Poisson error structure. We included individual identity as a random effect and assessed significance using likelihood-ratio tests. Next, we employed Non-metric Multidimensional Scaling (NMDS) running on strongylid community dissimilarities for ITS-2 variants, to infer between-sample differentiation. We used Adonis (i.e. permutations-based multivariate ANOVA for dissimilarity matrices) to test if there was any difference in strongylid community composition due to host species identity (i.e. gorilla vs. mangabey) and due to sample type (i.e. coprocultures vs. fecal samples). We specified individual identity as a permutation constraint to account for pseudo-replications.

We aligned reference ITS-2 sequences^33,34,36^ together with ITS-2 variants detected in our study using DECIPHER^65^ and constructed maximum-likelihood (ML) phylogenetic trees using R phangorn package^66^. We selected K80 + G^67^ as the best substitution model according to ModelTest^68^. We used bootstrapping analysis to assess the integrity of phylogenetic trees. We visualized distribution of ITS-2 variants in individual hosts in the phylogenetic context using ggtree^69^. As lengths of ITS-2 variants for *Necator* and *Oesophagostomum* differed considerably (349–359 bp and 241 bp respectively), we performed phylogenetic inference separately for these two genera. We rooted *Necator* phylogeny by *Ancylostoma duodenale* (NCBI accession: KC632570) and *Oesophagostomum* phylogeny by *Necator americanus* (NCBI accesion: AB793528) (Fig. S6). We performed all statistical analyses in R v. 3.1.0 (R Core Team 2014).

## Electronic supplementary material

Supplementary Information
