# Supplementary material for: Metabarcoding analysis of strongylid nematode diversity in two sympatric primate species
Source: Sci Rep. 2018 Apr 12;8:5933. doi: 10.1038/s41598-018-24126-3 (PMC5897349; doi:10.1038/s41598-018-24126-3)

Metabarcoding analysis of strongylid nematode diversity in two sympatric primate species

Barbora Pafčo*, Dagmar Čížková, Jakub Kreisinger, Hideo Hasegawa, Peter Vallo, Kathryn Shutt, Angelique Todd, Klára J. Petrželková, David Modrý

*Corresponding author

E-mail: [barafrikacar@gmail.com](mailto:barafrikacar@gmail.com)

**Supplementary information**

**S1 Fig.** Box-plots of read proportions corresponding to *Oesophagostomum* spp. in gorillas and mangabeys from DSPA.


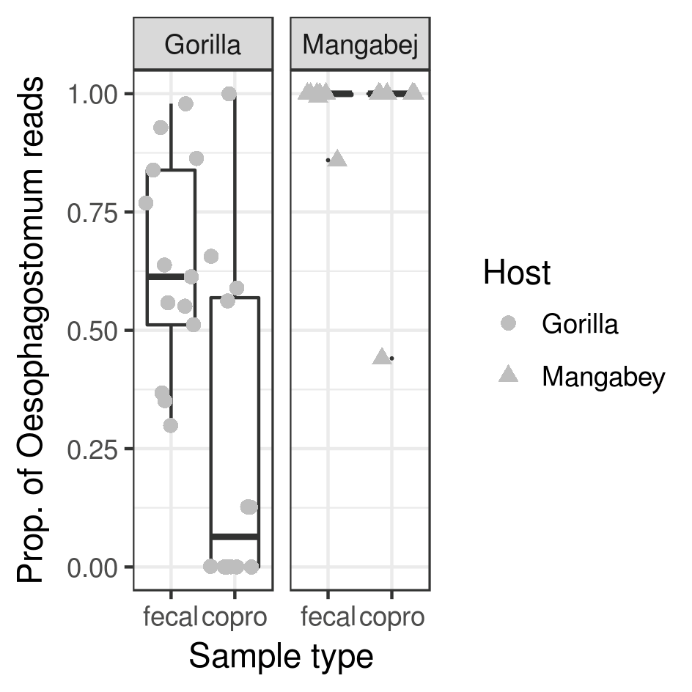


**S2 Fig**. ITS-2 sections available in GenBank corresponding to gene-specific primers and 10bp long 3’ flanking regions are shown. The rest of the ITS-2 sequences is denoted poly-N, due to high sequence length variation between strongylid genera.


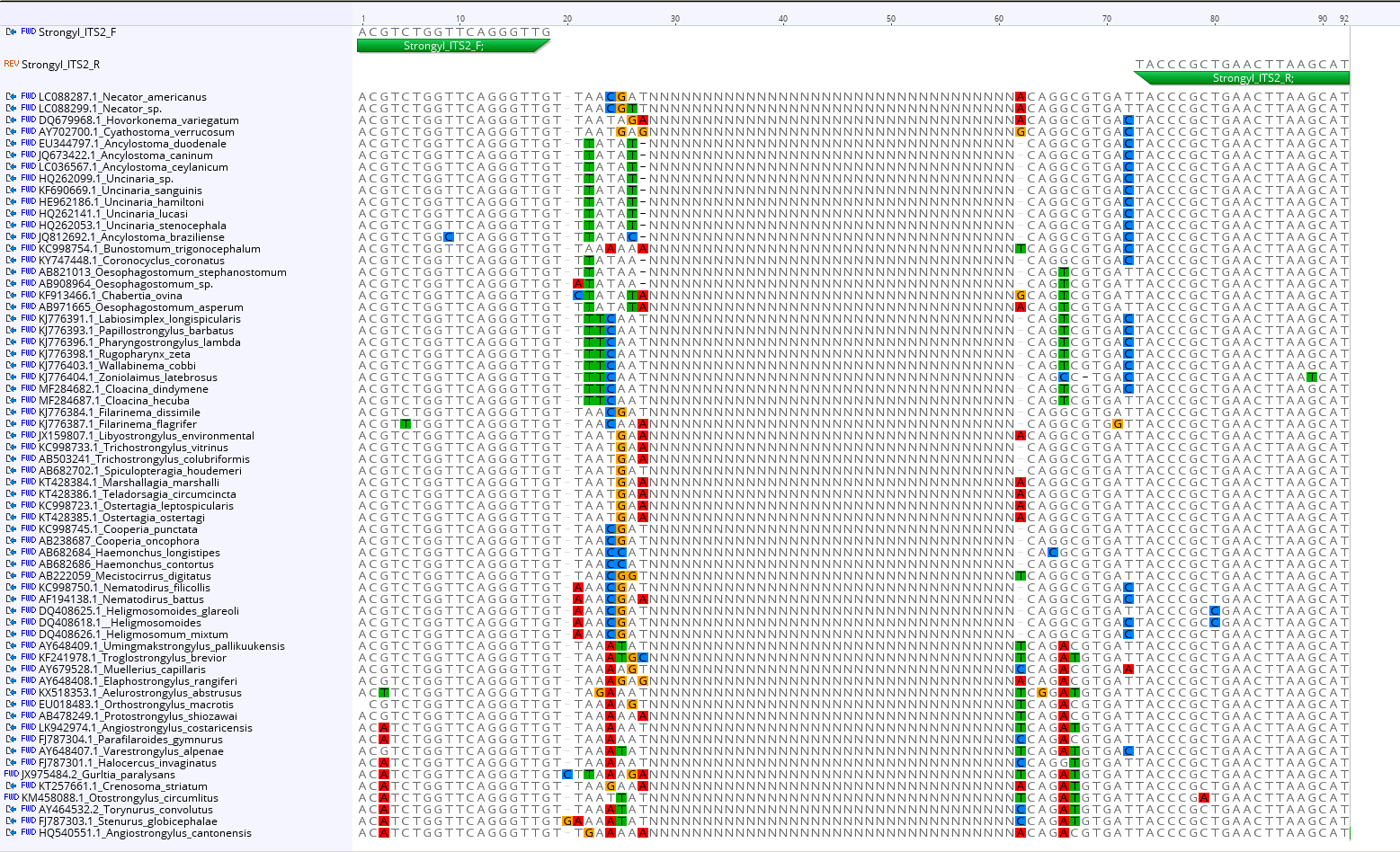


**S4 Appendix.** Synthetic DNA templates (Constructs)

Used as a positive control and to test for the biases (chimeric sequences, PCR errors, sequencing errors, contaminations) formed during the sample processing. Based on sequences available in GenBank, we created three sequence constructs (Construct 1-3), each including parts of 18S rDNA (primers in gray color), ITS-2 (primers in yellow) and *Cox*1 (primers in pink) of the strongylids species presumably present in great apes. For the purpose of later analyses, construct included 4bp tags (in red) not occurring in real ITS-2 haplotypes.

**Construct 1:** 18S_ *Necator* (AJ920348), ITS-2_*Necator americanus* (AB793527), Cox1_*Necator americanus* (AB793537)

CTCCACTAGTGTAAATCGTCATTGCTGCCCAAAAAAAGCTCGTAGTTGGATCTGAGTCGCATGCAGTGATTCGCCATTGGCGTTAATCGCTGTTGCGACTATTTGCTGGTTTTCTACTAAAGTTTCGGCTTTTTTAGTGGCTAGCGAGTTTACTTTGAATAAATTAGAGTGCTCAGAACAAGCGTTTGCTTGAATGCTCGATCATGGAATAATAAAAGAGGACTTCGGTTCTATTTATTGGTTCAGGAACTGAAATAATGGTTAAGAGGGACAATTCGGGGGCATTCGTATCCCTGCGCGAGAGGTGAAATTCGTGGACCGCAGGGGGACGCCCTAAAGCGAAAGCATTTGACGTCTGGTTCAGGGTTGTTAACGATAATAGATGAGTGTAGCTTGTGGACAGTACTCTCACCGAGTATTGTTGAACACTGTTTGTCGAACGGTACTTGCTCTGTACTACGCATTGTATACGTGTTCAGCAATTCCCGTTTAAGTGAAGAACACACGTGCAACATGTGCACGCTGTTATTCACTACGTTAGTTGGCTAGTTTACTAACGTATGATAGCGGTGCATACTGTATGACATGAACATATCGTTGTTCACTGTTTAATCGCTCTCGCGACTTATGAGCGTGGTTGAACGGAGACAATGTGAAGGACAACGATGTTCGCCATGTGGATGTGTCATTTGCAATGCAACCTGAGCTCAGGCGTGATTACCCGCTGAACTTAAGCATGGCTATTTTTAGTTTACATTGTGCTGGTTTAACAAGTATTTTAGGTGGTATTAATTTTATATGTACAACAAAAAATTTGCGTAGTAGATCTATTTCTTTAGAGCATATAAGTTTATTTGTGTGAACTGTGTTTGTTACGGTTTTTTTGTTGGTGTTGTCTTTACCAGTTTTGGCTGGAGCTATTACTATGTTGTTAACTGATCGTAATTTAAATACTTCTTTTTTTGATCCAAGTACTGGTGGTAATCCGTTAATTTATCAGCATTTGTTTTGATTTTTTGGGCATCCTGAGGTTTATATTTTAATTTTACCAGCATTTGGTATTATTAGGCAATCAACTTTATATTTAACTGGAAAAAAAGAAGTGTTTGGTTCTTTAGGTATAGTATATGCGATTTTAAGGATTGGTTTAATTGGTTGTGTAGTTTGAGCTCATCATATGTATACTGTTGGTATGGATTTGGATTCTCGAGCTTATTTTACTG

**Construct 2:** 18S_*Oesophagostomum* (AB677956), ITS-2_*Necator* sp. (AB793529), *Cox*1_*Necator* sp. (AB793568)

CTCCACTAGTGTAAATCGTCATTGCTGCAACCAAAAAGCTCGTAGTTGGATCTGAGTCACACGCAGTGGTTCGCCTTTGGCGTTAATCGCTGTTGTGACTATTTGCTGGTTTTCTACTAAGGTTTCGGCCTTTGTAGTGGCTAGCGAGTTTACTTTGAATAAATTAGAGTGCTCAGAACAGGCGTTTGCCTGAATGCTCGATCATGGAATAATAAAAGAGGACTTCGGTTCTATTTATTGGTTCAGGAACTGAAATAATGGTTAAGAGGGACAATTCGGGGGCATTCGTATCCCTGCGCGAGAGGTGAAATTCGTGGACCGCAGGGGGACGCCCTAAAGCGAAAGCATTTGACGTCTGGTTCAGGGTTGTTAACGTTATAAAGCTAGTGTAGCTTGTTACGACAATCATACTMATCGAGTGTTGTCGTGAACACTGTTTGTCGAACGGTACTTGTTCTAGCACATATTCATATGTGTTCAACAATTCCCGTTTAAGTGAAGAATAACACATGCAACATATGCAAACTGTCATTGTCAGTCACGTTAGTTGGCTAGTTTACTAACGCGCATATGGCATCATTGCATACTGTATGACATGAACATATCGTTGTTCACTGTTTAATCGCTCTAGCGACTTAAGAGTGTTTGAACGGAGACATGATGAAGGACAACGATGTTCACCAATTGTGGATGTGTCATTTGCAATGCAACCTGAGCTCAGGCGTGATTACCCGCTGAACTTAAGCATGGCTATTTTTAGTTTACATTGTGCTGGTTTAAACCTTATTTTAGGTGGTATTAATTTTATGTGTACTACTAAAAATTTGCGTAGGAGTTCTATTTCTTTGGAGCATATAAGTTTGTTTGTTTGAACGGTTTTTGTTACAGTCTTTTTGTTGGTTTTGTCTTTACCTGTTTTAGCTGGTGCTATTACTATGTTGTTAACTGATCGTAATTTAAATACTTCTTTTTTTGATCCTAGTACTGGTGGTAATCCTTTAATTTATCAACATTTGTTTTGATTTTTTGGGCATCCAGAGGTTTATATTTTGATTTTACCGGCTTTCGGTATTATTAGTCAGTCAACGTTATATTTAACGGGTAAAAAAGAAGTATTTGGTTCTTTAGGTATGGTGTATGCGATTTTGAGGATTGGTTTAATTGGTTGTGTAGTTTGAGCACATCATATGTATACTGTTGGAATGGATTTAGATTCTCGTGCTTATTTTACTG

**Construct 3:** 18S_*Ancylostoma duodenale* (EU344798), ITS-2_*Oesophagostomum stephanostomum* (KR149647), *Cox*1_*Oesophagostomum stephanostomum* (AB821034)

CTCCACTAGTGTAAATCGTCATTGCTGCTTGGAAAAAGCTCGTAGTTGGATCTGAGTCGCATGCAGTGGTTCGCCTTTGGCGTTAATCGCTGTTGCGACTATTTGCTGGTTTTCTACTGAAGTTTCGGCTTCTTTAGTGGCTAGCGAGTTTACTTTGAATAAATTAGAGTGCTCAGAACAAGCGTTTGCTTGAATGCTCGATCATGGAATAATAAAAGAGGACTTCGGTTCTATTTATTGGTTCAGGAACTGAAATAATGGTTAAGAGGGACAATTCGGGGGCATTCGTATCCCTGCGCGAGAGGTGAAATTCGTGGACCGCAGGGGGACGCCCTAAAGCGAAAGCATTTGACGTCTGGTTCAGGGTTGTTTATAAATATCCAAGTGTGGCTTGTGACGCTGTTTGTCGAACGATGCTTACATTTAGTGTGATCCTCGTTCTAGATGAGAAATATATTGCAACATGTATCTTGGTGCAATCCAAGAAGCACGGATGTCGTGACCTCGTTGTCACTGTCAAAGCGTTCAGCGACTAAGAATGCTTTGGCGGGGCCTGTATGACAACTGCGGTTTCATGTCATTTGCAATGCAACCTGAGCTCAGTCGTGATTACCCGCTGAACTTAAGCATAGCTATTTTTAGTCTTCATTGTGCAGGGTTGATCGATATTTTGGGGGGTATTAATTTTATGTGTACGACAAAAAATTTGCGTAGTAGATCTATTTCTTTGGAGCATATAAGTTTGTTTGTGTGAACTGTTTTTGTAACTGTATTTTTGTTGGTGCTATCATTACCTGTGTTGGCAGGGGCGATTACTATGTTATTAACTGATCGTAATTTAAATACTTCTTTTTTTGATCCTAGAACAGGGGGTAACCCATTAATTTATCAACATTTGTTTTGATTTTTTGGACATCCTGAGGTTTATATTTTGATTTTACCTGCGTTTGGGATTATTAGTCAGTCTACTTTGTATTTAACAGGTAAAAAAGAAGTTTTTGGGTCACTAGGTATGGTTTATGCAATTTTGAGGATTGGTTTAATTGGTTGTGTGGTGTGGGCTCACCACATGTATACAGTGGGTATAGATTTAGATTCTCGTGCTTATTTTACTG

**S5 Table:** The list of samples revealed from coprocultures and fecal samples from mangabeys and gorillas in DSPA and their accession numbers.

| **Sample id.** | **Individual id.** | **Sample type** | **Host** | **Accession.no** |
| --- | --- | --- | --- | --- |
| S9 | 32 | coproculture | gorilla | ERS1782700 |
| S10 | 33 | coproculture | gorilla | ERS1782625 |
| S12 | 47 | coproculture | mangabey | ERS1782626 |
| S13 | 48 | coproculture | mangabey | ERS1782627 |
| S14 | 50 | coproculture | mangabey | ERS1782628 |
| S15 | 51 | coproculture | mangabey | ERS1782629 |
| S16 | 103 | coproculture | gorilla | ERS1782630 |
| S17 | 109 | coproculture | gorilla | ERS1782631 |
| S18 | 123 | coproculture | gorilla | ERS1782632 |
| S19 | 128 | coproculture | gorilla | ERS1782633 |
| S20 | 149A | coproculture | gorilla | ERS1782634 |
| S21 | 171 | coproculture | gorilla | ERS1782635 |
| S22 | 172 | coproculture | gorilla | ERS1782636 |
| S23 | 188 | coproculture | gorilla | ERS1782637 |
| S24 | 189 | coproculture | gorilla | ERS1782638 |
| S25 | 21 | coproculture | gorilla | ERS1782639 |
| S26 | 195 | coproculture | mangabey | ERS1782640 |
| S27 | 196 | coproculture | mangabey | ERS1782641 |
| S28 | 197 | coproculture | mangabey | ERS1782642 |
| S33 | 32 | coproculture | gorilla | ERS1782643 |
| S34 | 33 | coproculture | gorilla | ERS1782644 |
| S36 | 47 | coproculture | mangabey | ERS1782645 |
| S37 | 48 | coproculture | mangabey | ERS1782646 |
| S38 | 50 | coproculture | mangabey | ERS1782647 |
| S39 | 51 | coproculture | mangabey | ERS1782648 |
| S40 | 103 | coproculture | gorilla | ERS1782649 |
| S41 | 109 | coproculture | gorilla | ERS1782650 |
| S42 | 123 | coproculture | gorilla | ERS1782651 |
| S43 | 128 | coproculture | gorilla | ERS1782652 |
| S44 | 149A | coproculture | gorilla | ERS1782653 |
| S45 | 171 | coproculture | gorilla | ERS1782654 |
| S46 | 172 | coproculture | gorilla | ERS1782655 |
| S47 | 188 | coproculture | gorilla | ERS1782656 |
| S48 | 189 | coproculture | gorilla | ERS1782657 |
| S49 | 21 | coproculture | gorilla | ERS1782658 |
| S50 | 195 | coproculture | mangabey | ERS1782659 |
| S51 | 196 | coproculture | mangabey | ERS1782660 |
| S52 | 197 | coproculture | mangabey | ERS1782661 |
| S57 | 32 | feces | gorilla | ERS1782662 |
| S58 | 33 | feces | gorilla | ERS1782663 |
| S59 | 46 | feces | gorilla | ERS1782664 |
| S60 | 47 | feces | mangabey | ERS1782665 |
| S62 | 50 | feces | mangabey | ERS1782666 |
| S63 | 51 | feces | mangabey | ERS1782667 |
| S64 | 103 | feces | gorilla | ERS1782668 |
| S65 | 109 | feces | gorilla | ERS1782669 |
| S66 | 123 | feces | gorilla | ERS1782670 |
| S67 | 149A | feces | gorilla | ERS1782671 |
| S68 | 128 | feces | gorilla | ERS1782672 |
| S69 | 171 | feces | gorilla | ERS1782673 |
| S70 | 172 | feces | gorilla | ERS1782674 |
| S71 | 188 | feces | gorilla | ERS1782675 |
| S72 | 189 | feces | gorilla | ERS1782676 |
| S73 | 32 | feces | gorilla | ERS1782677 |
| S74 | 33 | feces | gorilla | ERS1782678 |
| S75 | 46 | feces | gorilla | ERS1782679 |
| S76 | 47 | feces | mangabey | ERS1782680 |
| S78 | 50 | feces | mangabey | ERS1782681 |
| S79 | 51 | feces | mangabey | ERS1782682 |
| S80 | 103 | feces | gorilla | ERS1782683 |
| S81 | 109 | feces | gorilla | ERS1782684 |
| S82 | 123 | feces | gorilla | ERS1782685 |
| S83 | 149A | feces | gorilla | ERS1782686 |
| S84 | 128 | feces | gorilla | ERS1782687 |
| S85 | 171 | feces | gorilla | ERS1782688 |
| S86 | 172 | feces | gorilla | ERS1782689 |
| S87 | 188 | feces | gorilla | ERS1782690 |
| S88 | 189 | feces | gorilla | ERS1782691 |
| S89 | 21 | feces | gorilla | ERS1782692 |
| S90 | 195 | feces | mangabey | ERS1782693 |
| S91 | 196 | feces | mangabey | ERS1782694 |
| S92 | 197 | feces | mangabey | ERS1782695 |
| S93 | 21 | feces | gorilla | ERS1782696 |
| S94 | 195 | feces | mangabey | ERS1782697 |
| S95 | 196 | feces | mangabey | ERS1782698 |
| S96 | 197 | feces | mangabey | ERS1782699 |

**S6 Fig.** ML Phylogenetic tree including root position for A) *Necator* and B) *Oesophagostomum* ITS-2 haplotypes. Phylogenies were rooted by *Ancylostoma duodenale* and *Necator americanus*.


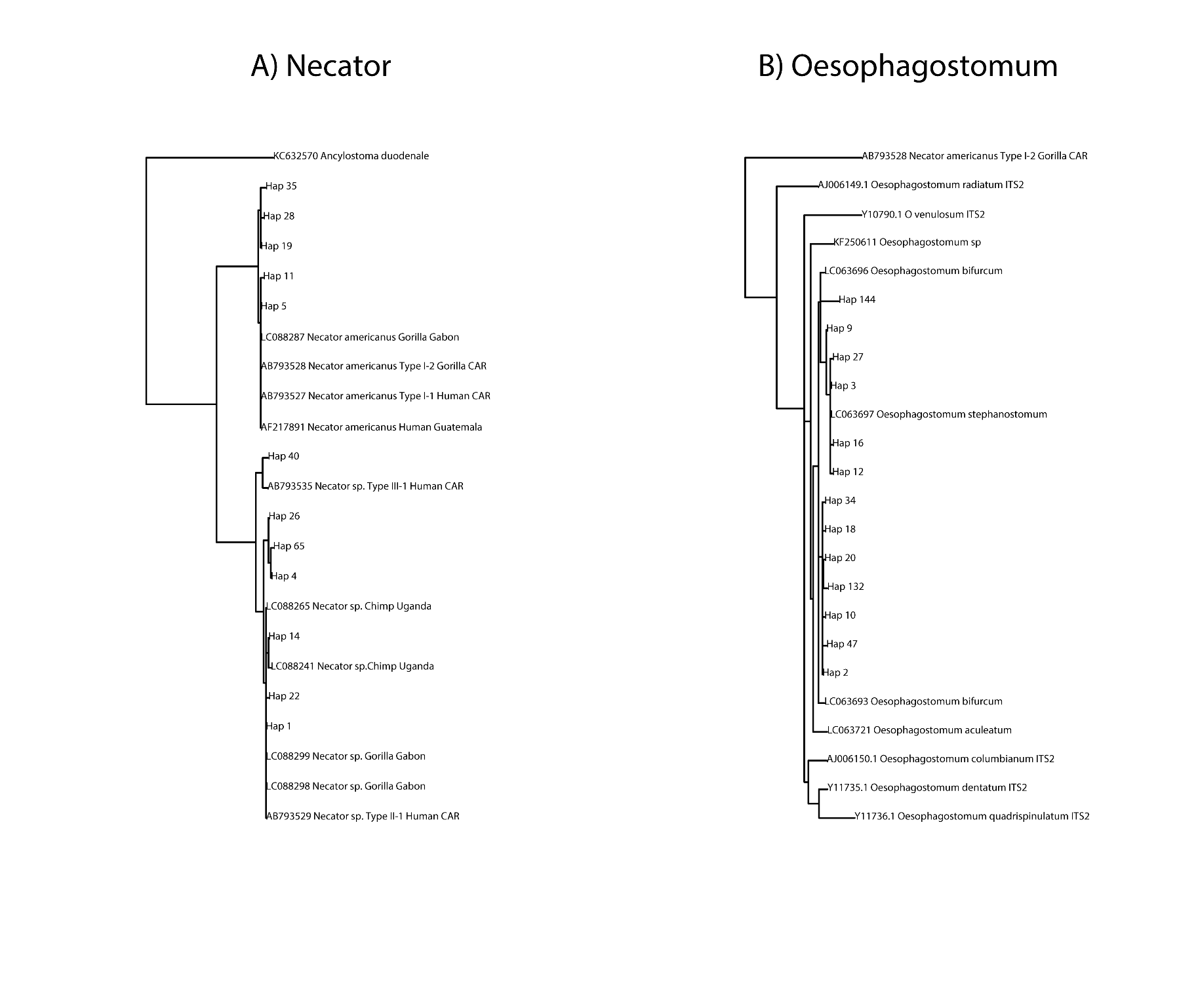

Supplement: Supplementary file 1 — Supplementary Information [file 41598_2018_24126_MOESM1_ESM.docx]
